# DEPENDS ON WHO YOU ASK: DISCORDANCE IN REPORTING SPOUSAL CARE BETWEEN WOMEN AND MEN ACROSS EUROPEAN WELFARE STATES

**DOI:** 10.1093/geroni/igae098.0638

**Published:** 2024-12-31

**Authors:** Afshin Vafaei, Ricardo Rodrigues, Cassandra Simmons, Eszter Zólyomi, Selma Kadi, Stefan Fors, Susan Phillips

**Affiliations:** Western University, London, Ontario, Canada; University of Lisbon, Lisbon, Lisboa, Portugal; European Centre for Social Welfare Policy & Research, Vienna, Wien, Austria; European Centre for Social Welfare Policy & Research, Vienna, Wien, Austria; European Centre for Social Welfare Policy & Research, Vienna, Wien, Austria; Karolinska institutet, Stockholm, Stockholms Lan, Sweden; Queen’s University, Kingston, Ontario, Canada

## Abstract

Current research on determinants, prevalence, and effects of informal care on health assumes that survey instruments can appropriately identify informal care providers. We aimed to investigate systematic differences across Europe in reporting spousal care between caregivers and cared-for persons and their possible effects on associations between informal care and physical and mental health indicators. Using information on care provided/received from the Survey on Health, Ageing and Retirement in Europe (SHARE), we estimated prevalence rates of spousal care and discordance between caregivers and cared-for persons in the reporting of care among caregiving dyads. We used multinomial regressions to estimate differences in reporting spousal care across European countries and then constructed multivariate logistic regression models to evaluate the effect of discordance in reporting informal care on the correlation between care and poor self-rated health and depression. Results showed that only 53.9% of dyads report care confirmed by both spouses. Multinomial regressions showed that agreement on care provided/received was more common when women are caregivers, while men were more likely to underreport providing/receiving care. There was no effect on the association of care with SRH regardless of who is asked to identify the care providers, while the magnitude and direction of the association between depression symptoms and care varies according to the choice of respondent. Possible explanations for the discordance include social norms dictating gender roles and acceptability of men as care providers. Relying on self-identification of care providers as most surveys do has the potential to underestimate their numbers.

